# Emerging fluconazole-resistant *Candida parapsilosis* in Australia: a case cluster and insights into the genetic diversity of this species

**DOI:** 10.3389/fmicb.2025.1742871

**Published:** 2026-01-21

**Authors:** Manoshi Perera, Winkie Fong, Rose Haywood, Geraldine J. Sullivan, Qinning Wang, Catriona L. Halliday, Sharn Dowsett-Moeahu, Chayanika Biswas, Carolina Firacative, Marie-Claire Liu, Kerry Weeks, Robyn Hardiman, Jen Kok, Kerri Basile, Wieland Meyer, Vitali Sintchenko, Sharon C-A Chen, Alice Kizny Gordon

**Affiliations:** 1 Mycology Reference Laboratory, Royal North Shore Hospital, New South Wales Health Pathology, St Leonards, NSW, Australia; 2 Clinical Mycology Reference Laboratory, Centre for Infectious Diseases and Microbiology Laboratory Services, Institute of Clinical Pathology and Medical Research, New South Wales Health Pathology, Westmead Hospital, Westmead, NSW, Australia; 3 Centre for Infectious Diseases and Microbiology – Public Health, Westmead Hospital, Westmead, NSW, Australia; 4 Sydney Infectious Diseases Institute, The University of Sydney, Camperdown, NSW, Australia; 5 Department of Microbiology, Royal Prince Alfred Hospital, New South Wales Health Pathology, Camperdown, NSW, Australia; 6 NSW Tissue Bank, NSW Organ and Tissue Donation Service, Incorporating the Lions NSW Eye Bank, NSW Bone Bank and the Australian Ocular Biobank, Sydney, NSW, Australia; 7 Commercial, Vaccines and Countermeasures Delivery, UK Health Security Agency, London, United Kingdom; 8 Studies in Translational Microbiology and Emerging Diseases (MICROS) Research Group, School of Medicine and Health Sciences, Universidad del Rosario, Bogota, Colombia; 9 Westerdjik Fungal Biodiversity Institute - KNAW, Utrecht, Netherlands; 10 Curtin Medical School, Faculty of Health Sciences, Curtin University, Perth, WA, Australia; 11 Sydney School of Veterinary Sciences, The University of Sydney, Sydney, NSW, Australia; 12 Northern Clinical School, Sydney Medical School, Faculty of Medicine and Health, University of Sydney, Sydney, NSW, Australia

**Keywords:** *Candida parapsilosis*, fluconazole-resistant *Candida* spp, nosocomial outbreak, outbreak investigation, whole genome sequencing

## Abstract

**Background:**

Nosocomial outbreaks of fluconazole-resistant *Candida parapsilosis* are concerning. Here we characterised a cluster of fluconazole-resistant *C. parapsilosis* utilising whole-genome sequencing (WGS) and correlate phenotypic azole resistance with resistome-based WGS analysis of azole resistance-conferring mutations.

**Materials and methods:**

Seventeen *C. parapsilosis* isolates were studied. Group 1: seven fluconazole-resistant isolates from a hospital intensive care unit (ICU) outbreak (2023–2024), Group 2: six isolates from a historical case cluster, Group 3: four additional unrelated isolates. Minimum inhibitory concentrations (MICs) were determined using SENSITITRE AUSNMRC1 (TREK Diagnostics). Single nucleotide polymorphism (SNP)-based phylogenomic analysis was undertaken using two (MycoSNP and custom-based) bioinformatic pipelines to assess relatedness. Target-gene mutations for azole resistance were evaluated.

**Results:**

ICU patient risks for fluconazole-resistant *C. parapsilosis* included presence of intravascular device and recent broad-spectrum antimicrobial use. Core SNP-based analysis showed clustering of Group 1, and separately, of Group 2 isolates. With greater genetic similarity (range <2–9 SNP difference) between isolates within Group 1, than between these and Group 2 and 3 isolates (1700–3,000 SNPs); the in-house pipeline yielded the same phylogenetic pattern with isolates within both Group 1 and 2 clusters separated by ≅100 SNPs (range 47–124). The eight fluconazole-resistant (MIC >64 mg/L) isolates had *ERG11* Y132F and *TAC1* D444Y mutations which were absent in fluconazole-susceptible isolates. The mutation *ERG11* R398I was present in azole-resistant and azole-susceptible strains.

**Conclusion:**

Genomic relatedness amongst clustered isolates was confirmed in this first fluconazole-resistant *C. parapsilosis* outbreak in Australia. Fluconazole-resistant isolates harboured *ERG11* Y132F and *TAC1* D444Y mutations. The protracted outbreak underscores the need to prioritise enhanced surveillance.

## Introduction

*Candida parapsilosis* is a significant pathogen, and is the second commonest cause of invasive candidiasis after *Candida albicans* in many regions (Pappas et al., 2018; Govrins and Lass-Flörl, 2024; World Health Organization, 2022; Daneshnia et al., 2023), while in recent years it has emerged as the most prevalent *Candida* species causing bloodstream infection in certain areas of South America, Southern Europe and South Africa (Sambo et al., 2025; Franconi et al., 2023; Shuping et al., 2021). It is concerning given its high mortality and association with nosocomial outbreaks, perpetuated by high transmissibility (Govrins and Lass-Flörl, 2024). These characteristics prompted the World Health Organization (WHO) to classify *C. parapsilosis* as a high-priority fungal pathogen (World Health Organization, 2022).

More recently, fluconazole-resistant strains of *C. parapsilosis* with minimum inhibitory concentrations (MICs) > 256 mg/L have emerged, with many countries documenting resistance rates exceeding 10% (Daneshnia et al., 2023). A South African paediatric study reported a resistance rate of 55% (Shuping et al., 2021), 17% in a nation-wide population based study within Kuwait (Alobaid et al., 2021), 35.3% in a single-centre study based in Korea (Kim et al., 2021), and 67.9% reported in a Brazilian oncology centre (Thomaz et al., 2021). These strains have been linked with endemicity, outbreaks in intensive care units (ICU) and high mortality (50–63.8%) (Chapman et al., 2017; Thomaz et al., 2018; Pinhati et al., 2016; Fekkar et al., 2021; Arastehfar et al., 2021). Patient risk factors include age <1 month, low birthweight, immunosuppression including transplantation and prior corticosteroid exposure, co-morbidities including malignancy, COVID-19 and diabetes mellitus, device-associated biofilm formation, presence of colostomy and use of total parenteral nutrition (Pappas et al., 2018; Govrins and Lass-Flörl, 2024; Daneshnia et al., 2023; Thomaz et al., 2018; Pinhati et al., 2016; Semet et al., 2025; Branco et al., 2023). Fluconazole resistance is primarily due to a mutation in the *ERG11* gene leading to the amino acid substitution Y132F (Daneshnia et al., 2023; Arastehfar et al., 2021), however other *ERG11* mutations, e.g., K143R, and mutations in genes encoding azole efflux pumps, e.g., *MRR1* and *TAC1,* may also confer resistance (Arastehfar et al., 2021; Doorley et al., 2022).

Outbreak analyses have been challenged by low resolution of standard molecular genotyping methods for distinguishing between *C. parapsilosis* strains (Tavanti et al., 2010). Newer approaches to assess genomic relatedness have included multi-locus sequencing typing (MLST) and microsatellite typing, with four studies utilising whole genome sequencing (WGS) (Pryszcz et al., 2013; Brassington et al., 2025; Sabino et al., 2010; Trobajo-Sanmartín et al., 2018; Misas et al., 2024; Daneshnia et al., 2023; McTaggart et al., 2024). Genome sequencing of three clinical and environmental isolates by Pryszcz *et al.* revealed unexpected genomic variability, although with low levels of heterozygosity between chromosomes (Pryszcz et al., 2013), while Brassington *et al*. reported outbreak strains were separated by ≅36 single nucleotide polymorphisms (SNPs) compared with a separation of >2,000 SNPs between unrelated strains (Brassington et al., 2025). Heterozygous SNPs may be expected in diploid fungi such as *C. parapsilosis* (genome size 13 Mbp) (Wang et al., 2023). Therefore, SNP variation and epidemiologically relevant thresholds defining genetically-related clusters require better understanding (McTaggart et al., 2024).

The prevalence of fluconazole-resistant *C. parapsilosis* in Australia is unknown but expected to be low (1.2%) based on a previous snap-shot surveys (Chapman et al., 2017). In 2023–2024 however, fluconazole-resistant *C. parapsilosis* clinical isolates emerged for the first time over 13 months in the ICU of a large Sydney tertiary hospital. This prompted the present study, which sought to determine the epidemiological links between the patients. By WGS, we also studied the genetic diversity amongst the current potential “outbreak” isolates, compared with isolates independent of the outbreak, including those from a separate historical putative case cluster. We further placed the WGS sequences in a global context and compared phenotypically azole-resistant isolates with azole-susceptible isolates for mutations in *ERG11* and other genes including *TAC1* and *MRR1* implicated in antifungal resistance.

## Materials and methods

### Definitions

As per the Australian Guidelines for the Prevention and control of Infection in Healthcare, this study defined an outbreak as there was an occurrence of more cases of fluconazole-resistant *C. parapsilosis* infection than expected in a specific group of patients in a given area over a particular period of time (National Health and Medical Research Council, 2019).

### Isolates and sequences

Seventeen *C. parapsilosis* isolates were studied and assigned to three groups (Table 1).

**Table 1:** Details of 17 *Candida parapsilosis* isolates studied in this report.

Patient label	Isolate ID	Date or year of isolation	Body site of isolation	Fluconazole-resistant (Yes/No)
Group 1 (Royal North Shore Hospital)				
Patient A	23–008-0007	22/03/2023	Arterial line tip	Yes
Patient B	23–008-0008	10/03/2023	Wound swab (deep)	Yes
Patient C	24–008-0008	10/08/2023	Blood	Yes
	24–008-0009	14/08/2023	Catheter tip	Yes
Patient D	24–008-0010	19/9/2023	Tissue	Yes
Patient E	24–008-0012	30/03/2024	Sputum	Yes
Patient F	24–008-0013	1/3/2024	Urine	Yes
Group 2* (Lions NSW Eye Bank)				
Patient 1	WM 17.12	2017	Eye (anterior chamber)	No
Patent 2	WM 17.13	2017	Eye (anterior chamber)	No
Patient 3	WM 17.14	2017	Eye (anterior chamber)	No
Patient 4	WM 17.16	2017	Eye (anterior chamber)	No
Patient 5	WM 17.31	2017	Eye (anterior chamber)	No
Patient 6	WM 17.68	2017	Eye (anterior chamber)	No
Group 3 (other “control” isolates)				
	WM 01.216	2021	Blood	No
	WM 02.200	2022	Blood	No
	WM 09.77	2009	Quality Control strain (originally from blood)	No
	23–008-0004	2023	Genital (vagina)	Yes

ID, Identification; NSW, New South Wales. *Isolated over a 5-month period from April to August 2017.

Group 1 comprised seven fluconazole-resistant isolates from the current potential outbreak, from six patients who were either infected or colonised, in the ICU, Royal North Shore Hospital, Sydney, Australia, between March 2023 and April 2024. Group 2 encompassed six isolates representing a historical cluster, obtained from the culture collection at the Department of Microbiology, Institute of Clinical Pathology and Medical Research, donated by the New South Wales (NSW) Tissue Bank, Sydney. Group 3 included four unrelated or “control” isolates not linked to Group 1 or 2 clusters, also from the aforementioned culture collection.

Isolates were identified to species by MALDI-TOF MS (Bruker Biotyper v4.2, Bruker Daltonics, Bremen, Germany) and by sequencing of the internal transcribed spacer region (Clinical and Laboratory Standards Institute, 2018). All isolates were *C. parapsilosis sensu stricto.* All were subcultured for purity prior to study.

#### Clinical context of group 1 isolates

Seven fluconazole-resistant *C. parapsilosis* (MIC ≥8 mg/L) isolates were recovered from six ICU patients from various body sites (Table 1; Clinical and Laboratory Standards Institute, 2022). Following identification of the first two isolates in March 2023, routine antifungal susceptibility testing was performed on all *C. parapsilosis* isolates from ICU patients to enhance surveillance but dedicated patient, healthcare worker and environmental screening for *C. parapsilosis* was not performed. Routine antifungal susceptibility testing was not performed prior to this on *Candida* isolates from non-sterile sites, in keeping with local institutional guidelines, as *Candida* species are common skin colonisers and further testing is only performed on clinically significant isolates and upon clinician requests. The patients were temporo-spatially in the same ICU ward space linked over 13 months. The ICU comprised four adjacent 15-bed wards, with each bed in a single room and access to a shared common central area.

Patient data were extracted from electronic medical records including demographics, risk factors for *C. parapsilosis* infection, microbiological findings and geographic transition of patients during admission. Patients received antifungal therapy through standard institutional protocols. All-cause mortality and overall global response outcomes at 30 days were defined (Segal et al., 2008). Human Research Ethics Committee approval was obtained (HREC identifier ETH02179).

#### Group 2 isolates

These six fluconazole-susceptible isolates were part of a historical cluster, independent of Group 1 isolates. Clinical details of this cluster cannot be disclosed for legal reasons, however all cases were isolated within a 5-month period in 2017 from a single healthcare facility, where all patients had received care in a common hospital area. Permission has been given by the NSW Tissue Bank, Organ & Tissue Donation Service (Mr. Sharn Dowsett-Moeahu; Supplementary Document S1) to utilise the sequences of these isolates to compare with those of Group 1 and 3 to infer relatedness. All data are de-identified.

### Antifungal susceptibility testing

Susceptibility testing was performed using the SENSITITRE AUSNMRC1 (TREK Diagnostics, Cleveland, OH, United States) plates as per manufacturer instructions. Nine antifungal agents were tested with drug dilution ranges: amphotericin B (0.12–8 mg/L), anidulafungin (0.015–8 mg/L), micafungin (0.008–8 mg/L), 5-flucytosine (0.06–64 mg/L), isavuconazole (0.008–8 mg/L), posaconazole (0.008–8 mg/L), voriconazole (0.008–8 mg/L), itraconazole (0.015–16 mg/L) and fluconazole (0.12–256 mg/L). Quality control strains were *Pichia kudriavzevii* ATCC 6258 and *C. parapsilosis* ATCC 22019. MICs were determined after incubation at 35 °C for 24 h and interpreted in reference to the clinical and laboratory standards institute (CLSI) clinical breakpoints (Clinical and Laboratory Standards Institute (CLSI), 2022). Fluconazole resistance was defined as a MIC ≥8 mg/L and voriconazole resistance, a MIC ≥1 mg/L. (Clinical and Laboratory Standards Institute, 2022).

### DNA extraction, PCR amplification and sequencing

Isolates were grown on Sabouraud’s dextrose agar and incubated at 35 °C for 48 h. Genomic DNA extraction was performed using the MasterPure™ Yeast DNA purification kit (Epicentre, Lucigen Corporation, United States). DNA concentration was quantified using the Quant-iT™ PicoGreen™ dsDNA assay kit (Life Technologies, Carlsbad, CA). Genomic libraries were constructed using Illumina DNA prep (Illumina, San Diego, CA) and sequenced on the NextSeq 500 instrument with NextSeq 500/550 v2 Mid-Output kits (Illumina) with 2× 150 bp paired-end chemistry.

### WGS analysis

Raw reads of the sequence data were passed through an in–house quality control procedure, including assessment of read quality and cross-contamination using Trimmomatic v0.36 with default parameters (Bolger et al., 2014), FastQC v0.11.3 and Centrifuge v1.0.4-beta. Minimum read coverage of ≥40X after trimming was accepted for all isolates. Trimmed reads were mapped to the reference genome of *C. parapsilosis* strain CDC 317^1^ to ensure consistency with annotations previously described (Skrypek et al., 2017).

Since the approach to utilising SNP-based phylogenomic analysis by WGS in *C. parapsilosis* is relatively new, and that data to assess genetic relatedness in this species are relatively few, we used two approaches to conduct the analysis, namely to capture any heterozygous SNPs which arise due to the diploid nature of *C. parapsilosis* genome. The first comprised of identifying whole-genome SNPs from raw FASTQ sequences using the MycoSNP pipeline (Bagal et al., 2022). FASTQ reads were mapped to a repeat-masked reference genome of *C. parapsilosis* CDC317 using the Burrows-Wheeler Alignment (BWA) Tool v07.17-r1188 and de-duplication was performed using Picard MarkDuplicates v2.20.6 as contained within MycoSNP (Skrypek et al., 2017; Butler et al., 2009). Variant calling was performed with the Genome Analysis toolkit (GATK) HaplotypeCaller v4.2.5.0 and variants filtered using GATK VariantFiltration. SNP distances were called using SNP-dists v0.8.2.^2^ Only biallelic SNPs with no missing sites were retained. Core genome similarity between isolates was assessed. The phylogenetic tree was drawn using FastTree v2.1.10 and visualised with ggtree v3.14.0 (Price et al., 2009; Yu et al., 2016). A cluster was arbitrarily defined as a group of isolates with <20 SNPs difference (McTaggart et al., 2024).

For the second approach, an in-house custom pipeline was followed. Reads were mapped to an unmasked version of the same reference genome as above to compare SNP distances and clustering thresholds (Supplementary Document S2). The pipeline retained heterozygous SNPs for inclusion in genomic interrogation for potential genetic mechanisms of antifungal resistance. A phylogenetic tree was constructed with IQ-TREE v2.2.2 using a GTR + G4 model (Minh et al., 2020).

All genomic data are shared under the BioProject number PRJNA1301451. Australian *C. parapsilosis* genomes were placed in global context and compared with 115 genome sequences downloaded from the National Centre for Biotechnology Information (NCBI) Sequence Read Archive (SRA).

### Assessment of antifungal drug resistance markers

Reads were mapped to genes associated with antifungal resistance using BWA v0.7.13 (Li and Durbin, 2009). The target genes included *ERG11*, *ERG3, FKS1*, *FKS2*, *MRR1*, *TAC1* and *UPC2* (Arastehfar et al., 2021; Branco et al., 2023; McTaggart et al., 2024). A consensus sequence was generated from the BAM file using SAMtools v1.16 (Li et al., 2009). The sequence was translated to its amino acid representation using EMBOSS v6.6.0.0. The consensus sequence was converted to a VCF file with SNP-sites v2.3.3 to automate the extraction of amino acid changes relative to the wild-type (Page et al., 2016).

### Other investigations

Whole-genome copy number variant (CNV) analysis and mating type locus (MTL) investigation was performed with DELLY CNV v.0.8.7 applied to the VCF files generated from MycoSNP with a ploidy flag of 2 (Rausch et al., 2012). Finally, we searched for loss of heterozygosity (LOH) events across *ERG11* using a custom R script (Berkow and Lockhart, 2017). For each variant position, frequencies were calculated across a 10,000 bp window, with a region considered as LOH if the rate of heterozygosity consistently dropped below 0.1%.

## Results

### ICU *Candida parapsilosis* cluster (group 1)

The ICU comprised four adjacent 15-bed wards, with each bed in a single room and access to a shared common central area. The six patients (Table 1; Supplementary Table S1) were distributed amongst these four wards. Each patient changed room during their ICU admission 2–4 times, with 2 of 6 also changing wards. Three patients occupied the same room over disparate timepoints. Median time from hospital admission to positive culture was 34.0 days (interquartile range, IQR 26–56) while the median time from ICU admission to culture positivity was 21.0 days (IQR 10–23.5). There was a male preponderance (66.6%) with median age 63.5 years (IQR 60.8–67.8). Patient risk factors included haematological malignancy (2/6, 33.3%), recent prior antifungal use (5/6, 83.3%) including half of the patients with prior azole use, and an intravascular catheter (6/6, 100%). Two patients were colonised while four had infections; of the latter, two had favourable clinical outcomes following echinocandin therapy. Half the patients died within 30 days. Interventions to limit further transmission included patient isolation, initiation of contact transmission-based precautions, single-use equipment, regular quaternary ammonium disinfection of shared equipment and terminal cleaning of departed rooms with 1000 ppm sodium hypochlorite (NaOCl) solution and vapourised 6% hydrogen peroxide (H_2_O_2_). Susceptibility testing of *C. parapsilosis* isolates cultured from ICU patients beyond the study period did not identify further fluconazole-resistant strains.

### Susceptibility profiles of *Candida parapsilosis* (groups 1–3)

MICs of all studied isolates are shown in Table 2. For Group 1 isolates, fluconazole MICs were all ≥64 mg/L, with one (strain 24–008-0013) having an MIC of 256 mg/L; this isolate also had a voriconazole MIC of 8 mg/L, while the other six isolates had voriconazole MICs between 1 and 2 mg/L. The MICs to itraconazole, posaconazole and isavuconazole were ≤0.5 mg/L. Group 2 isolates had low MICs to all antifungal drugs, with fluconazole MICs ≤2 mg/L. Of the remaining isolates only one, strain 23–008-0004, exhibited a high fluconazole MIC (128 mg/L), though with a voriconazole MIC of 1 mg/L and low MICs to the other triazoles and echinocandins. Thus, there were eight fluconazole-resistant isolates. There were no echinocandin-resistant isolates. MICs to amphotericin B (<0.12–1.0 mg/L) and 5-flucytosine (<0.06–0.12 mg/L) were all wild-type (Clinical and Laboratory Standards Institute, 2022).

**Table 2:** *In vitro* susceptibilities of 17 *Candida parapsilosis* isolates to nine antifungal agents and major amino acid substitutions relative to wild-type isolate sequences identified in genes associated with resistance to fluconazole, where present.

Isolate ID	MIC (mg/L)									Gene and mutations	
	AMB	ANID	MICA	5FC	ISAV	POS	VRC	ITC	FLU	*ERG11*	*TAC1*
Group 1 (Royal North Shore Hospital)											
23–008-0007	0.50	1.0	2.0	<0.06	0.25	0.25	2.0	0.25	64*	Y132F, R398I	D444Y
23–008-0008	1.0	1.0	1.0	<0.06	0.25	0.25	1.0	0.25	64*	Y132F, R398I	D444Y
24–008-0008	<0.12	0.50	1.0	<0.06	0.25	0.06	1.0	0.12	64*	Y132F, R398I	D444Y
24–008-0009	<0.12	0.50	1.0	<0.06	0.25	0.06	1.0	0.12	64*	Y132F, R398I	D444Y
24–008-0010	0.50	0.50	1.0	<0.06	0.12	0.12	1.0	0.25	64*	Y132F, R398I	D444Y
24–008-0012	1.0	1.0	2.0	0.12	0.008	0.50	8.0	0.50	256*	R398I	–
24–008-0013	0.50	1.0	1.0	<0.06	0.50	0.12	2.0	0.12	64*	Y132F, R398I	D444Y
Group 2 (Lions NSW Eye Bank)											
WM 17.12	0.50	0.50	0.50	<0.06	0.008	0.008	0.03	0.03	1.0	–	–
WM 17.13	0.50	0.50	1.0	0.06	0.015	0.03	0.03	0.03	1.0	–	–
WM 17.14	0.50	0.50	0.50	<0.06	0.008	0.008	0.015	0.015	1.0	–	–
WM 17.16	0.50	0.25	1.0	0.06	0.06	0.06	0.06	0.06	2.0	–	–
WM 17.31	0.25	0.25	0.50	<0.06	0.008	0.008	0.008	0.015	1.0	–	–
WM 17.68	0.25	0.50	0.50	<0.06	<0.008	<0.008	<0.008	0.060	1.0	–	–
Group 3 (other “control” isolates)											
WM 01.216	0.50	0.50	0.50	<0.06	<0.008	0.015	0.008	0.03	0.50	R398I	–
WM 02.200	0.25	0.50	0.50	<0.06	<0.008	<0.008	<0.008	0.06	1.0	–	R208G
WM 09.77	0.25	0.50	0.50	<0.06	<0.008	<0.008	<0.008	0.06	1.0	–	–
23–008-0004	1.0	0.50	0.50	<0.06	0.06	0.03	1.0	0.12	128*	Y132F	G490R, D444Y

AMB amphotericin B; ANID anidulafungin; FLU fluconazole; 5-FC 5-flucytosine; ID identification; ISAV isavuconazole; ITC itraconazole; MIC minimum inhibitory concentration; MICA micafungin; NSW New South Wales; POS posaconazole; VRC voriconazole. *Fluconazole resistant (Clinical and Laboratory Standards Institute (CLSI), 2018).

### Genomic features of sequenced *Candida parapsilosis* isolates

Isolates were sequenced to an average depth of 76-168X (data not shown). Analysis of the MTL revealed that all isolates sequenced were homozygous for the MTLa idiomorph, with MTLa2 present and no detectable MTLα genes. There was no LOH.

#### Phenotypic and genotypic resistance correlations

There were clear differences in mutation profiles between fluconazole-resistant and fluconazole-susceptible strains. Of fluconazole-resistant isolates, mutations in the *ERG11* gene leading to the amino acid substitution Y132F were evident in all Group 1 isolates except for isolate 24–008-0012 (Table 2). Other mutations identified were *ERG11* R398I and *TAC1* D444Y. The fluconazole-resistant isolate 23–008-0004 (Group 3) also contained the *ERG11* Y132F and *TAC1* D444Y mutations. None of the fluconazole-susceptible isolates carried either of *ERG11* Y132F or *TAC1* D444Y mutations; however, one (strain WM01.216) harboured the *ERG11* R398I mutation, and another (strain WM02.200), the *TAC1* R208G mutation. There were no mutations identified in the genes *ERG3, MRR1* and *UPC2* in the studied isolates nor were there non-synonymous polymorphisms in the *C. parapsilosis FKS1* or *FKS2* genes.

CNV analysis indicated increased copy number of *ERG11* in several isolates, including Group 1 strains (23–008-0007, 23–008-0008, 24–008-0008, 24–008-0009, 24–008-0010, 24–008-0013), Group 2 strains (WM17.12, WM17.16) and a Group 3 strain (23–008-0004).

### Genomic relatedness between *Candida parapsilosis* strains (groups 1–3)

**Figure 1:**
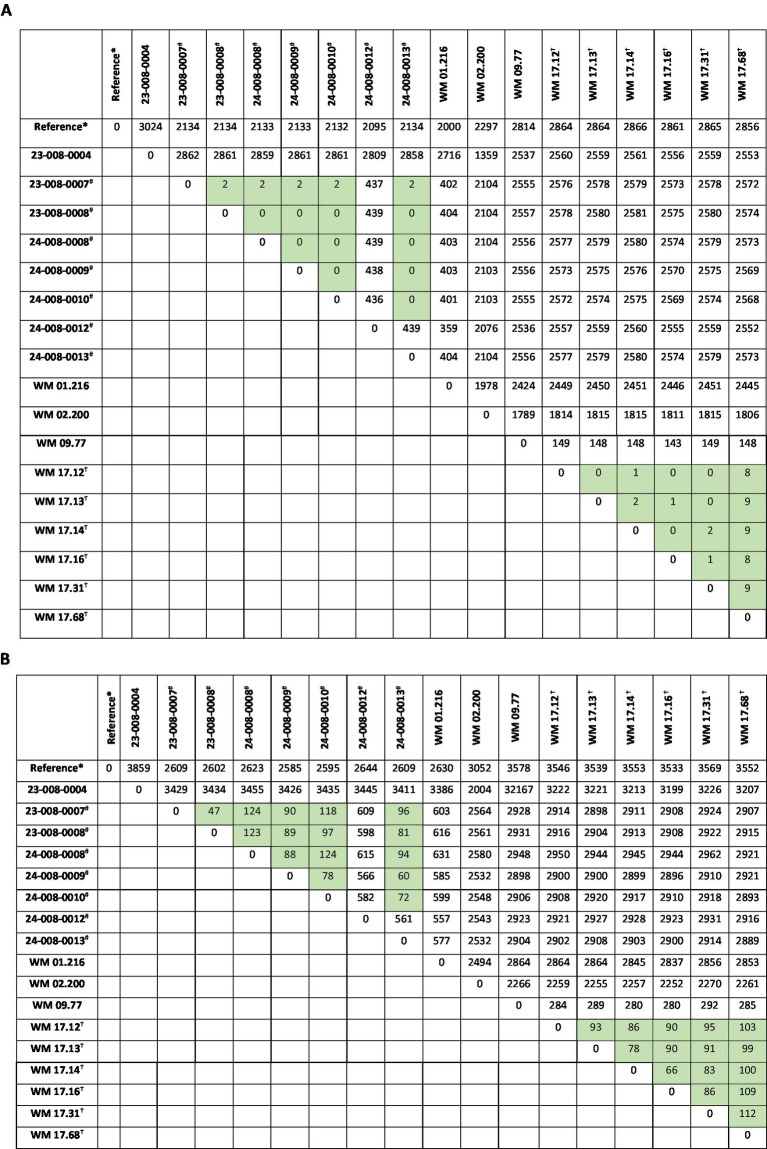
**(A)** Pairwise SNP matrix between clinical *Candida parapsilosis* isolates, determined via WGS and analysed using MycoSNP. Each cell represents the number of SNP differences between the corresponding isolates. The green shading highlights the SNP differences between the two clusters of isolates. **(B)** Pairwise SNP differences between clinical *Candida parapsilosis* isolates, determined via WGS and analysed using an in-house custom pipeline (Supplementary Document S2). Each cell represents the number of SNP differences between the corresponding isolates. The green shading highlights the SNP differences between the two clusters of isolates. *Reference genome is *C. parapsilosis* strain CDC 317 (http://www.candidagenome.org/download/sequence/C_parapsilosis_CDC317; last accessed 2nd October 2025). ^#^Denotes isolates of Group 1 (strains 23–008-0007, 23–008-0008, 24–008-0008, 24–008-0009, 24–008-0010, 24–008-0012, 24–008-0013). ^Ϯ^Denotes isolates of Group 2 (strains WM 17.12, WM 17.13, WM 17.14, WM 17.16, WM 17.31, WM 17.68).

Core genome comparison of 18 sequences (17 studied isolates, and reference *C. parapsilosis* CDC317 strain) using the MycoSNP pipeline, revealed two clusters (Figure 1A). One cluster comprised five sequences from the ICU outbreak (Group 1 isolates), and the second cluster included five Group 2 isolates. These two clusters were separated from each other and from sequences of other unrelated isolates; in general, the genomes differed by 1,700–3,000 SNPs.

Group 1 isolates were genetically similar (0–2 SNP differences), except for isolate 24–008-0012 which yielded >400 SNP differences with the others but remained distinct from Group 2 and 3 isolates (Figure 1A). Of the Group 3 isolates however, strain WM01.216 was more closely related to isolate 24–008-0012 by approximately 400 SNPs compared with 1000s of SNP differences with other isolates in the group. The second cluster amongst Group 2 isolates also displayed minor differences (0–9 SNPs) between isolates. These were distinct from the sequences of Group 1, and most Group 3 isolates, but more similar to that of isolate WM09.77.

The analysis of genomic similarity using an in-house pipeline, without masking repetitive regions of the *C. parapsilosis* reference genome, reproduced the same clustering pattern, but with larger estimates of the genomic distance between isolates, i.e., isolates within a cluster differing at a threshold of ≅100 SNPs (range 47–124). Unrelated isolates were separated by 2000–3500 SNPs. Isolate 24–008-0012 was the least related within Group 1 (Figure 1B). Amongst Group 2 isolates, these were least divergent to the Group 3 isolate WM09.77.

### Phylogenetic analysis

**Figure 2:**
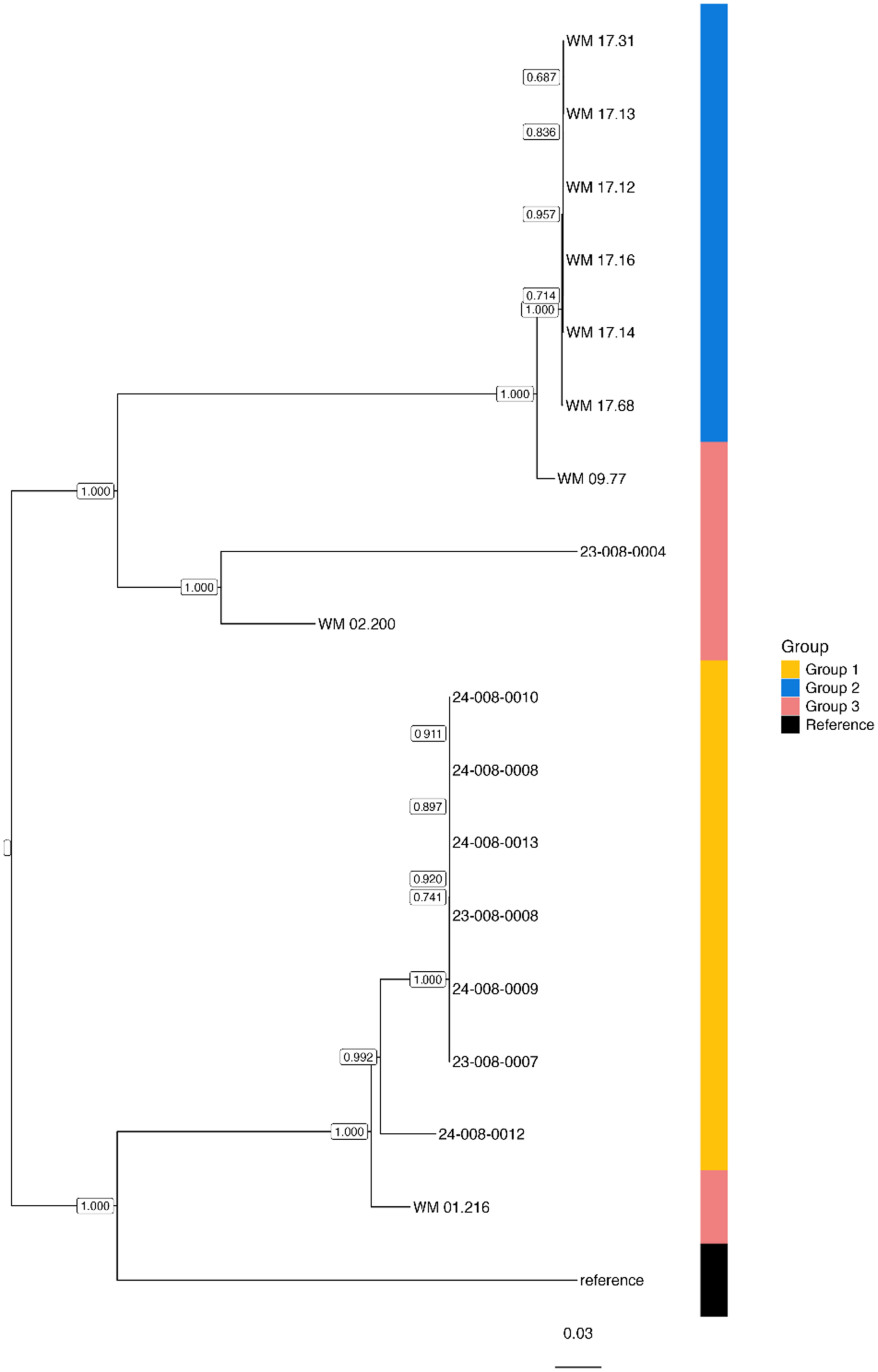
Maximum-likelihood phylogeny of *Candida parapsilosis* genomes generated in this study based on genome-wide SNPs using MycoSNP. Bootstrap values are shown next to internal nodes. Genomes are directly annotated as tip labels, and the adjacent heatmap corresponds to the two distinct clusters derived from separate outbreaks. *Reference genome is *C. parapsilosis* strain CDC 317 (http://www.candidagenome.org/download/sequence/C_parapsilosis_CDC317; last accessed 2nd October 2025).

The core SNP-likelihood phylogenetic analysis of *C. parapsilosis* isolates (Figure 2) confirms the relationship inferred from the SNP matrix, showing clear clustering of isolates in Group 1 and 2. Within Group 1, isolate 24–008-0012 falls outside the main cluster but remains closely related. The phylogeny also highlighted greater genetic similarity between isolate WM09.77 (Group 3) and the cluster of Group 2 isolates, and between isolate WM 01.216 and the cluster of Group 1 isolates. The phylogenetic tree generated by the in-house pipeline showed the same clustering and genetic relationships (data not shown). Both pipelines produced identical clusters with high (100%) bootstrap support and indicated two subgroups within Group 1 isolates. There was no apparent correlation between the subgroup and patient location.

**Figure 3:**
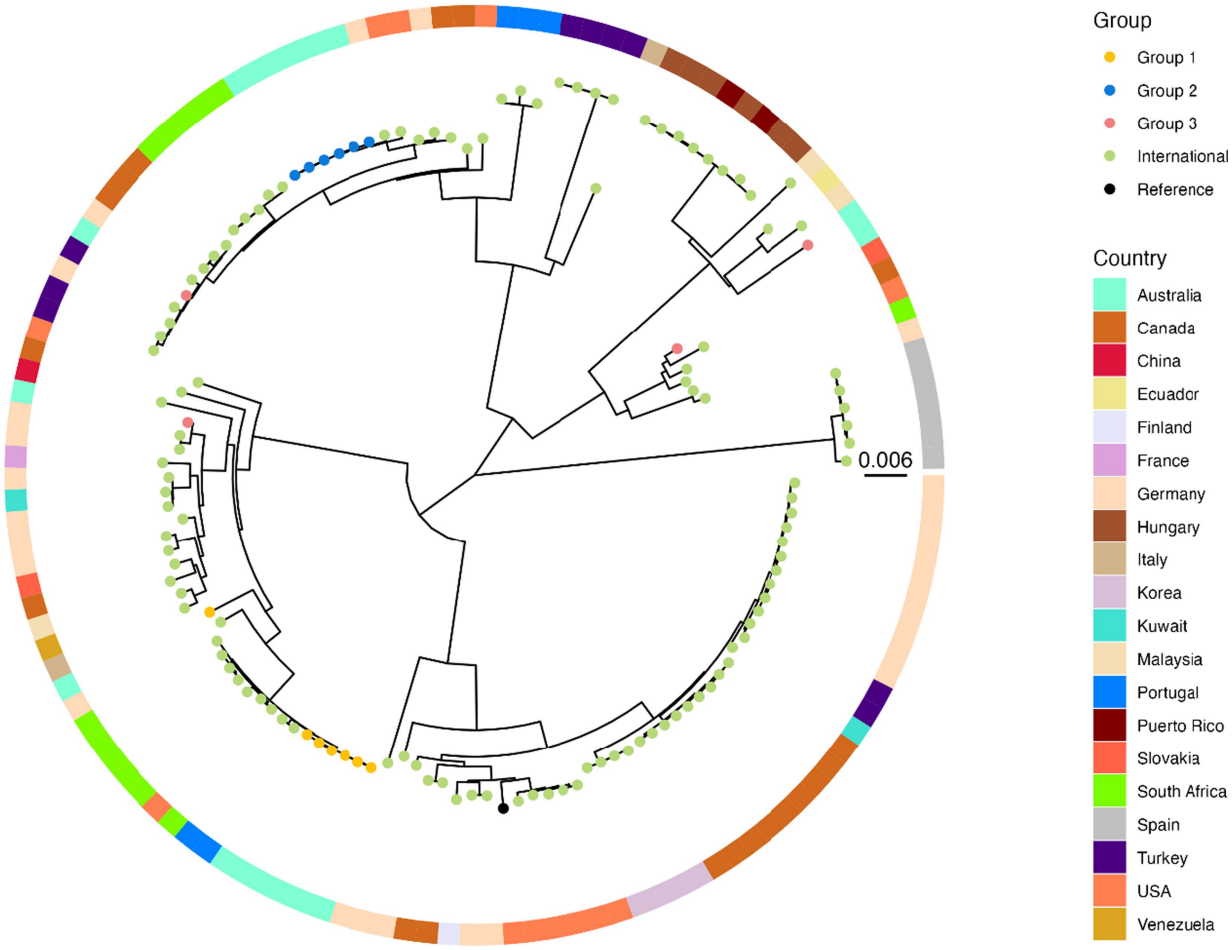
Maximum-likelihood phylogenetic tree of *Candida parapsilosis* genomes, including isolates sequenced in this study and publicly available SRA samples (*n* = 115), constructed using genome-wide SNPs and analysed with MycoSNP. Branch lengths represent the number of nucleotide substitutions per site. Tip colors indicate assigned groups as described in the legend, and the surrounding ring represents the country of origin for each isolate, with colors corresponding to the countries shown in the heatmap legend. *Reference genome is *Candida parapsilosis* strain CDC 317 (http://www.candidagenome.org/download/sequence/C_parapsilosis_CDC317; last accessed 2nd October 2025).

Phylogeny of the Australian isolates within a global context demonstrates that the Group 1 and 2 genomes from this study form distinct clusters, with no international genome occupying the same branch. Nevertheless, this analysis highlighted the difference between strain 24–008-0012 and the rest of the Group 1 isolates and demonstrated that several international isolates are more closely related to our clusters. Two isolates from Germany and the United States of America (USA) appear similar to the Group 2 isolates, while several isolates from South Africa and Portugal show closer genetic similarity to the Group 1 cluster (Figure 3). Genomes allocated to Group 3 were dispersed amongst international isolates.

## Discussion

*Candida parapsilosis* has received increasing attention as a pathogen due to emergence of azole-resistant strains (Thomaz et al., 2018; Arastehfar et al., 2021; Escribano and Guinea, 2022). Here we describe for the first time, an outbreak of fluconazole-resistant *C. parapsilosis* in an Australian ICU which was limited by heightened infection control practices adapted from *Candidozyma auris* (formerly *Candida auris*) management guidelines (see Results) (CEC Auris Guidelines, 2025). Other key findings include the support for an outbreak detection provided by WGS in which two bioinformatic pipelines identified greater genetic similarity (range <9–≅100 SNP difference) between isolates within this cluster than between unrelated isolates (2000–3500 SNP difference). Our results also highlight implications for managing azole-resistant *C. parapsilosis* and reinforce the case for using WGS to genotype this species.

Outbreaks of fluconazole-resistant *C. parapsilosis* have varied in duration and have often occurred in ICU settings (Thomaz et al., 2018; Pinhati et al., 2016; Escribano and Guinea, 2022; Presente et al., 2023). Furthermore, prompt investigation of any disease from baseline prevalence is required as outlined by Australian national and local guidelines in outbreak investigations and management (National Health and Medical Research Council, 2019). Our ICU cluster was small but highlighted a definitive increase in cases from baseline prevalence in a localised area and over a defined period of 13 months. No definitive source of infection or mode of transmission identified. This was similar to the experience of Fekkar et al. (2021), where an epidemiological link was difficult to establish due to a small cohort size, disparate time intervals between cases and transmission leading to colonisation rather than infection. In contrast, Thomaz et al. (2018), identified intravascular catheters, while others demonstrated healthcare worker hands, as likely sources for transmission (Clark et al., 2004; Qi et al., 2018). Since we had not previously detected fluconazole-resistant *C. parapsilosis* isolates in our facility, we hypothesised transmission between patients. Utilising WGS, isolates within the Group 1 cluster demonstrated greater genomic similarity to each other (Figures 1A,B) than to those not epidemiologically linked. A SNP difference of <10 separated isolates from the ICU cluster (MycoSNP analysis), and genomic relatedness was replicated using an in-house pipeline without masking repetitive regions (<47–124 SNP difference). Both analyses produced the same overall clustering with some Group 1 isolates more closely clustered than Group 2 isolates (Figure 2). Our findings are concordant with a multi-centre Canadian case cluster study in which WGS by MycoSNP analysis (using a cut-off of <20 SNPs) showed greater similarity between outbreak isolates than an in-house pipeline (McTaggart et al., 2024). A German study demonstrated a SNP cut-off value of 36 (SD 20) but SNP analysis methodology was not stated (Brassington et al., 2025), while another study estimated the SNP cut-off to vary from <10 to >200 (Guinea et al., 2022).

Of interest, previous studies reported fluconazole-susceptible isolates showed greater SNP differences than fluconazole-resistant strains. This has been ascribed to the heightened capacity of resistant strains for clonal spread, and potential fitness advantage for environmental persistence (Daneshnia et al., 2023; Fekkar et al., 2021; Misas et al., 2024). In our study however, there was little evidence to suggest that drug resistance influences genetic variation. Fluconazole-susceptible isolates from the Group 2 cluster were more genetically similar to one another compared with epidemiologically unrelated isolates.

Phylogenetic analysis reiterated the distinct clustering of azole-resistant isolates from Group 1 compared with Group 2 and 3 isolates. Interestingly, isolate 24–008-0012 was more distantly related compared with other Group 1 isolates, with a unique resistance gene pattern and the highest fluconazole MIC, despite being the penultimate case. This may indicate the presence of fluconazole-resistant *C. parapsilosis* strains in the environment prior to the study period, or reflect a more complex transmission pathway, that we were unable to identify. The latter has been highlighted previously, where the lack of high-resolution data on patient transfers may not capture the role that reintroducing pathogens plays in perpetuating outbreaks (Brassington et al., 2025). As the first fluconazole-resistant *C. parapsilosis* outbreak that has undergone genomic evaluation in Australia, global contextualisation helped emphasise the distinct nature and unique clustering of the studied isolates. However, relatedness suggesting shared evolutionary relationships with strains circulating internationally was noted between isolates from Group 1 and geographically-disparate countries, including South Africa and Portugal. This likely reflects the global endemicity and rising prevalence of fluconazole-resistant *C. parapsilosis* (Daneshnia et al., 2023; Brassington et al., 2025).

Gene mutations associated with fluconazole resistance were identified in all eight azole-resistant isolates. Substitution of Y132F in the *ERG11* gene, associated with ergosterol biosynthesis, is the most frequently cited mutation, and was identified in all but one resistant isolate (strain 24–008-0012) (Branco et al., 2023). Another shared *ERG11* mutation, R398I, although described in combination with other drug resistance mutations, was also found in azole-susceptible isolates (Choi et al., 2018). Furthermore, we did not observe the *ERG11* K134R mutation either alone or in combination with Y132F which is known to confer azole-resistance (Arastehfar et al., 2020), nor other *ERG11* mutations, e.g., G458S, K128N (Arastehfar et al., 2021). CNV analysis demonstrated an increased copy number of *ERG11* in several phenotypically-resistant isolates, which may contribute to elevated azole MICs (Ning et al., 2024), and has been observed in *C. auris* (Muñoz et al., 2018). The MTL analysis yielded the homozygous state, consistent with reports that most clinical *C. parapsilosis* isolates are homozygous at this locus and rarely undergo sexual reproduction (Bergin et al., 2022).

Notably, our study identified a common *TAC1* gene mutation, D444Y, not previously described to confer fluconazole resistance. *TAC1* encodes a transcription factor which, if mutated to a gain-of-function mutation such as G650E, leads to overexpression of the Mdr1 efflux pump and fluconazole resistance (Hartuis et al., 2021). The only isolate in our study without the *TAC1* D4447 mutation had significantly higher azole MICs (isolate 24–008-0012), suggesting a loss-of-function mutation. Further investigation is required to delineate the impact of this mutation on azole resistance. There were no *MRR1* (Doorley et al., 2022), *UCP2* or *CDR1* mutations (Branco et al., 2023; Kim et al., 2022).

Factors proposed to increase risk of acquiring fluconazole-resistant *C. parapsilosis* include diabetes mellitus, and pharmacological immunosuppression (Thomaz et al., 2018; Pinhati et al., 2016) - as was observed in our study. Although half of the Group 1 patients received prior azoles, there has been insufficient evidence to suggest that such use increases the risk of fluconazole-resistant *C. parapsilosis* (Fekkar et al., 2021; Arastehfar et al., 2021). Studies have demonstrated increasing resistance rates paralleling rising fluconazole use in healthcare facilities over time (Thomaz et al., 2018; Arastehfar et al., 2021). Other risk factors common to all Group 1 patients included prolonged hospitalisation prior to their positive culture, presence of intravascular catheter and recent broad-spectrum antimicrobials. The significance of the latter was previously described in bone marrow transplant recipients on prophylactic antibiotics, where proliferation of *C. parapsilosis* occurred in the intestinal microbiota with attributable reduction in survival (Daneshnia et al., 2023). Identification of azole-resistant strains also has implications for clinical practice guidelines including antifungal prophylaxis for high-risk groups (Keighley et al., 2021).

This study was limited by the small number of isolates analysed, and the lack of active surveillance via patient, healthcare worker and environmental screening. Potential additional cases including colonisation may have been missed. However, routine patient screening for *Candida* is not typically recommended and would pose microbiological and economic challenges given the prevalence of *C. parapsilosis* in skin microbiota. The lack of environmental isolates is a limitation as knowledge of the natural genomic variability of isolates within the hospital environment is fundamental to contextualising strain relatedness in any phylogenetic investigation (Litvintseva et al., 2015). More in depth heterozygosity and LOH analyses may have provided more clarity in interpretation of relatedness between strains.

In conclusion, we present the first fluconazole-resistant *C. parapsilosis* outbreak in an Australian healthcare facility and verified the genomic relatedness of the clustered isolates by WGS using two complementary approaches. The findings also confirmed a correlation between phenotypic azole-resistance and the resistome predictions derived from whole-genome analysis. Genomic similarity of the isolates despite the protracted duration of the outbreak highlights ongoing challenges for infection control and the need for enhanced surveillance.

## Data Availability

The datasets presented in this study can be found in online repositories. The names of the repository/repositories and accession number(s) can be found in the article/Supplementary material.

## Footnotes

1 http://www.candidagenome.org/download/sequence/C_parapsilosis_CDC317, Last accessed 2nd October 2025.

2
https://github.com/tseemann/snp-dists
