# Supplementary material for: Emerging fluconazole-resistant Candida parapsilosis in Australia: a case cluster and insights into the genetic diversity of this species
Source: Front Microbiol. 2026 Jan 21;16:1742871. doi: 10.3389/fmicb.2025.1742871 (PMC12871536; doi:10.3389/fmicb.2025.1742871)
Supplement: Supplementary file 1 [file Data_Sheet_1.pdf]

Supplementary files

Supplementary Document 1: Supplementary methods

Reads were mapped using BWA v07.17-r1188.[39] Bam files were sorted and indexed using SAMtools v1.6,[40] and deduplication performed using Picard v2.20.6 MarkDuplicates with default parameters.[59] SNPs were predicted using FreeBayes v1.3.2 and default parameters.[60] To exclude low coverage sites from the studied isolates, a bedfile containing the union of all low coverage positions across the genome was produced using a custom R script. Positions demonstrating elevated heterozygosity (>5 heterozygous positions within 50 bp of each other and uninterrupted by SNPs and lower than average read depth for the chromosome) were also appended to the low coverage bedfile. A consensus sequence for each sequence was then constructed from the VCF file, including only SNPs and excluding variants intersecting with coordinates in the bedfile described above. The individual sequences were combined into a multi-FASTA file and SNP-sites v2.3.3,[37] as used to exclude monomorphic genomic positions. Pair-wise SNP distances were then calculated using SNP-dists v0.8.2 with the '-a' flag employed to include IUPAC ambiguous positions in the comparisons. For the phylogenetic comparison, genomic positions where at least one sequence in the cohort exhibited low coverage were excluded.

Supplementary Table 1: Demographic and clinical characteristics of six patients in the intensive care unit from whom seven fluconazole-resistant isolates were cultured (Group 1 isolates).

**Isolate ID**	**23-008-0007**	**23-008-0008**	**24-008-0008**	**24-008-0009**	**24-008-0010**	**24-008-0012**	**24-008-0013**
Time from hospital admission to positive culture (days)	54	22	30	34	14	58	66
Time from ICU admission to positive culture (days)	31	22	21	25	11	8	9
**Patient label**	**A**	**B**	**C**		**D**	**E**	**F**
Age	79	35	60		69	64	63
Gender	Female	Female	Male		Male	Male	Male
**Risk Factors**							
Diabetes mellitus	No	No	No		Yes	Yes	No
Haematological malignancy	Yes	No	Yes		No	No	No
Intravascular catheter device	Yes	Yes	Yes		Yes	Yes	Yes
Pharmacological immunosuppression*	Yes	No	Yes		No	Yes	Yes
Recent broad-spectrum antimicrobial use	Yes	Yes	Yes		Yes	Yes	Yes

Recent antifungal use	Yes	Yes	Yes	No	Yes	Yes
Recent triazole use	Yes	No	Yes	No	No	Yes
Colonisation vs infection	Infection	Infection	Infection	Colonisation	Colonisation	Infection
**Clinical management and outcome**						
Directed echinocandin therapy for *C. parapsilosis*	No	Yes	Yes	No	Yes	Yes
Clinical outcome**	Death	Complete response	Complete response	NA	NA	Death
All-cause 30-day mortality	Yes	No	No	No	Yes	Yes

Abbreviations: ICU intensive care unit; ID identification; NA not applicable.

*Pharmacological immunosuppression including corticosteroid therapy use, systemic chemotherapy or other immunosuppressive therapy.

**Clinical outcome as defined by Segal *et al.*[31]
